# Supplementary material for: miR‐132 loss de‐represses ITPKB and aggravates amyloid and TAU pathology in Alzheimer's brain
Source: EMBO Mol Med. 2016 Aug 2;8(9):1005–18. doi: 10.15252/emmm.201606520 (PMC5009807; doi:10.15252/emmm.201606520)
Supplement: Supplementary file 1 — Appendix [file EMMM-8-1005-s001.pdf]

Appendix

Table of contents

Appendix Table S1. Primers used in the study

Appendix Table S1. Primers used in the study

**Primer (mRNAs)**	**Sequence (5' - 3')**
Sirt1 Forward	TAATCAGGTAGTTCCTCGGT
Sirt1 Reverse	CTCACTTTCAGAGAAGATCCA
Hspa2 Forward	CATCATCAATGAGCCCACAG
Hspa2 Reverse	TCTTGTGTTTGCGCTTGAAC
Taf4 Forward	GGCTCCAGGAACACCTA
Taf4 Reverse	AGCTGTGGCTGTTCCAA
Kiaa1958 Forward	ACTAAGCTCAACAAATTTCCTG
Kiaa1958 Reverse	GATGTCCGTGTGGTCTT
Arid1a Forward	TCCCAGCAAACTGCCTATTC
Arid1a Reverse	GGGCCTTGACTGAAGACTCA
Zbtb34 Forward	GCGTGTCTGAGTATGAGATT
Zbtb34 Reverse	TCAACCATCTCCGTGTGGTA
Zcchc11 Forward	CCGACTTGAAAAACGGTCAC
Zcchc11 Reverse	TTAAAGCAGCCAAGTGAGCA
Arhgap21 Forward	CAGAGTCTGCAATCCAGT
Arhgap21 Reverse	TTTATAATTCTGTCACCTGTGCAT
Kcnn3 Forward	TTTGGAATTGTTGTTATGGTGATAGA
Kcnn3 Reverse	GCTGTACTTCCCTTGTGT
Pard3 Forward	CTGGATACAGGTGCATCG
Pard3 Reverse	GAATATCTCTGGACTCTGGGT
Znf395 Forward	GAGCCACAGATAGAAGT
Znf395 Reverse	TCCTGACCTCCATACCA
Erbb2ip Forward	GCTCTATTTAGATGCTAATCAGATTGA
Erbb2ip Reverse	CATTTTTGCTGACATCCAGT
Prpf4b Forward	AAGAGCGGAAGTCAAAACGA
Prpf4b Reverse	TCATTGAGAAGCGGAGACCT
Capn2 Forward	CCTCACCTTGAATGAGGAAAT
Capn2 Reverse	CACAAAGAGCAGCTCCC
Itpkb Forward	GACCAAGCCACGTTACA
Itpkb Reverse	CACTTGAAGAAGGGAGAGATTT
Sp3 Forward	GTAGCTTGCACCTGTCC
Sp3 Reverse	CCACAGAACATCCAGTTACAAATA
Itgb8 Forward	TCTCTGGAAACATAGACACCC
Itgb8 Reverse	TTTCCGTCATTCGGCAC

Nfib Forward	GGAGTCAACTTCCCAATCG
Nfib Reverse	GTAGGACTTGGCTCCATATTT
Tjp2 Forward	GAGACGTCAATCGTCATCTCA
Tjp2 Reverse	CTGCTATCTTCCCACTTTTCCTAA
Actin Forward	TCCTCCCTGGAGAAGAGCTA
Actin Reverse	GCAATGATCTTGATCTTC
Gapdh Forward	TTGATGGCAACAATCTCCAC
Gapdh Reverse	CGTCCCGTAGACAAAATGGT
**Primer (small RNAs)**	**TARGET SEQUENCE**
mmu-miR-132	UAACAGUCUACAGCCAUGGUCG
mmu-miR-212-3p	UAACAGUCUCCAGUCACGGCCA
mmu-miR-127-3p	UCGGAUCCGUCUGAGCUUGGCU
mmu-miR-29b-3p	UAGCACCAUUUGAAAUCAGUGUU
