# Supplementary material for: miR‐132 loss de‐represses ITPKB and aggravates amyloid and TAU pathology in Alzheimer's brain
Source: EMBO Mol Med. 2016 Aug 2;8(9):1005–18. doi: 10.15252/emmm.201606520 (PMC5009807; doi:10.15252/emmm.201606520)
Supplement: Supplementary file 2 — Expanded View Figures PDF [file EMMM-8-1005-s002.pdf]

Expanded View Figures

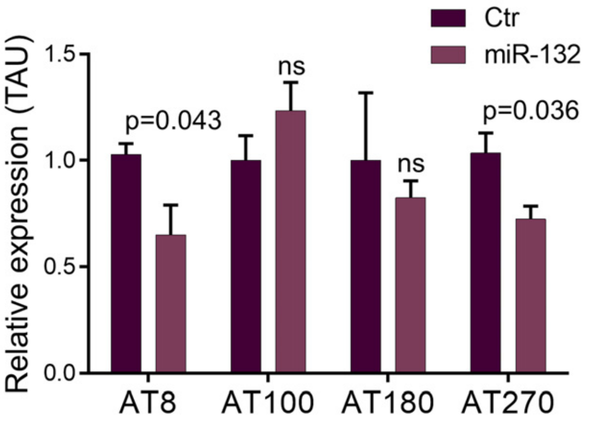

Phospho epitope Antibody	Ser202/ Thr205 AT8	Thr212/ Ser214 AT100	Thr231 AT180	Thr181 AT270
ERK1/2	+	-	-	+
GSK3b	+	+	+	+
CDK5	+	+	+	+

Figure EV1. Differential regulatory effect of miR-132 on TAU phospho-epitopes.

Quantification of Western blot analysis in APPPS1 hippocampal lysates with antibodies recognizing distinct phosphosites on TAU upon miR-132 overexpression (miR-132) at 3 months of age. Sample size, $n = 6$ per group. Values were normalized to control-injected group and presented as mean ± SEM. Student's t-test was used. An overview of the effects on distinct TAU phosphosites is given in the table provided.

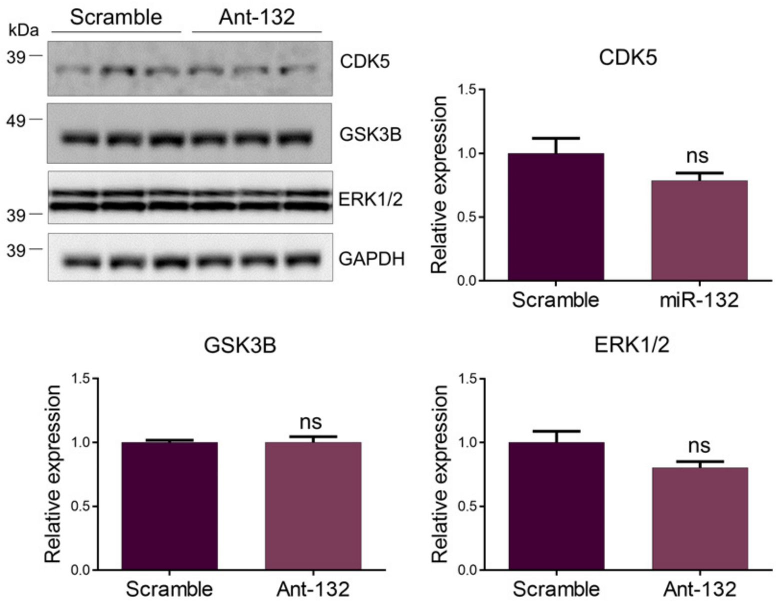

Figure EV2. Expression levels of TAU kinases upon miR-132 downregulation.

Western blot analysis of CDK5, GSK3B and ERK1/2 in ant-132-injected mice at 6 months of age. Sample size, $n = 9$ per group. Values were normalized to scramble-injected group and presented as mean ± SEM. Student's t-test was used.

Source data are available online for this figure.

© 2016 The Authors

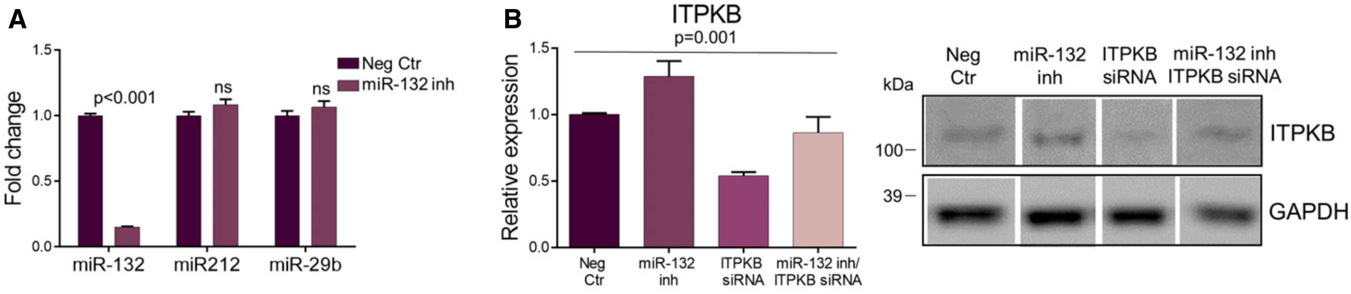

Figure EV3. Knockdown efficiency of miR-132 and ITPKB *in vitro*.

A Semi-quantitative PCR of miR-132 and control miRNAs in HEK293-APP^{swe} cells transfected with a miR-132 antisense inhibitor (miR-132 inh) or a negative control oligonucleotide (Neg Ctr).

B Western blot analysis of ITPKB levels in HEK293-APP^{swe} cells transfected with miR-132 antisense oligonucleotide (miR-132 inh), an siRNA against ITPKB (ITPKB siRNA) or both.

Data information: Sample size in (A and B), n = 3 (three independent experiments, each in triplicates). Values were normalized to the respective control groups and presented as mean ± SEM. Student's t-test was used in (A), while in (B), one-way ANOVA was employed.
Source data are available online for this figure.

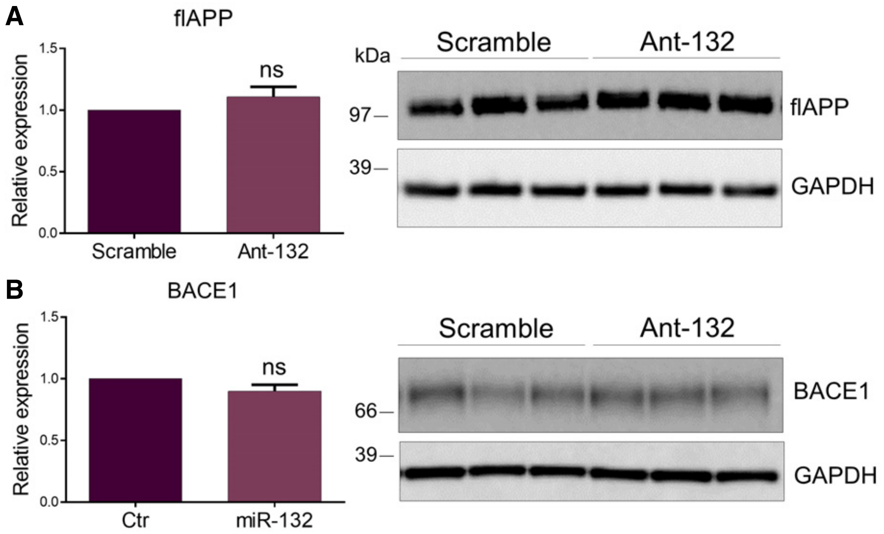

Figure EV4. Effect of miR-132 downregulation on APP and BACE1 expression.

A, B Western blot analysis of full length APP (A) and BACE1 (B) levels in ant-132-injected mouse hippocampus at 6 months of age. Sample size, n = 9 per group. Values were normalized to scramble-injected groups and presented as mean ± SEM. Student's t-test was used.

Source data are available online for this figure.

 © 2016 The Authors

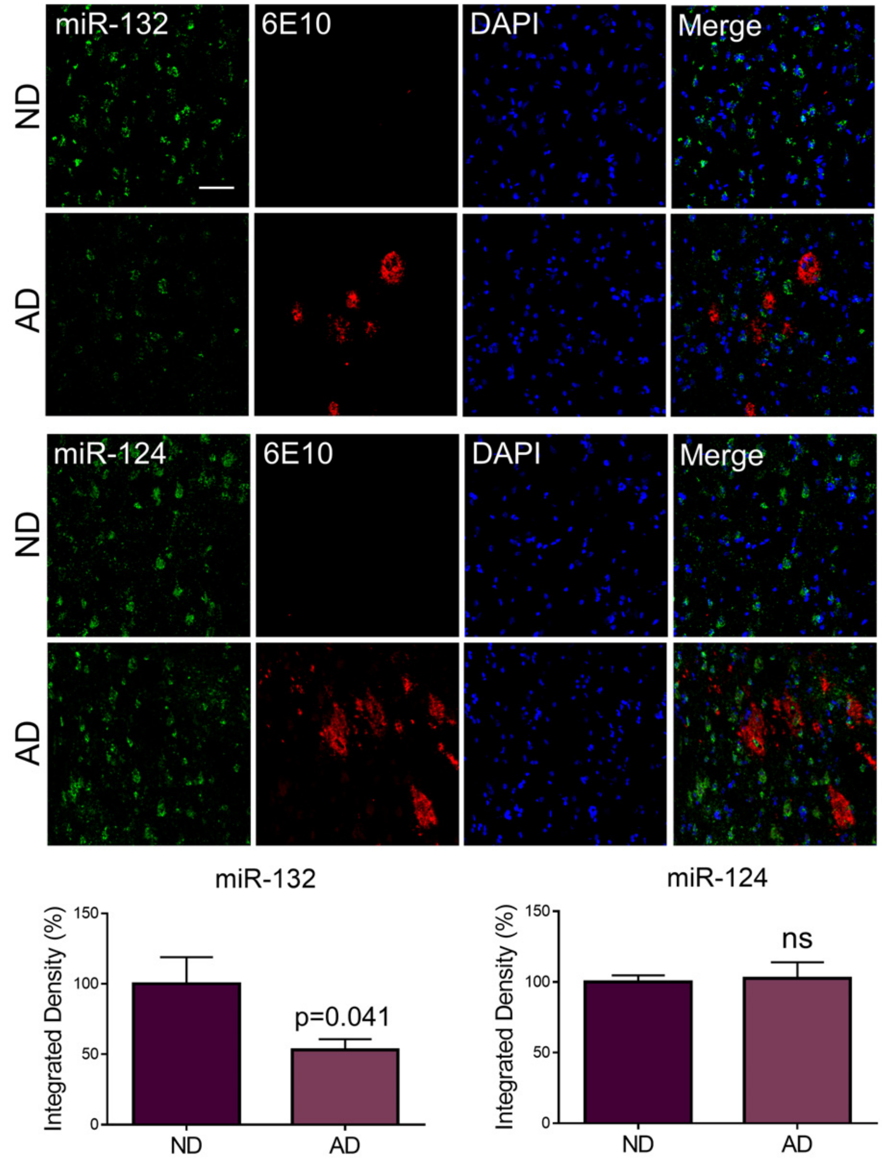

Figure EV5. miR-132 deficiency in human AD prefrontal cortex.
FISH of miR-132 and negative control miR-124 in prefrontal cortex from AD patients (AD) and non-demented (ND) controls combined with amyloid immunostaining (6E10). Graphs represent quantification of the percentage of integrated density (see Materials and Methods). Sample size, n = 3 per group. Values were normalized to ND samples and presented as mean ± SEM. Student's t-test was used. Scale bar, 500 μm.

© 2016 The Authors
