# Supplementary figures and images for: miR‐132 loss de‐represses ITPKB and aggravates amyloid and TAU pathology in Alzheimer's brain
Source: EMBO Mol Med. 2016 Aug 2;8(9):1005–18. doi: 10.15252/emmm.201606520 (PMC5009807; doi:10.15252/emmm.201606520)

**Figure EV2**

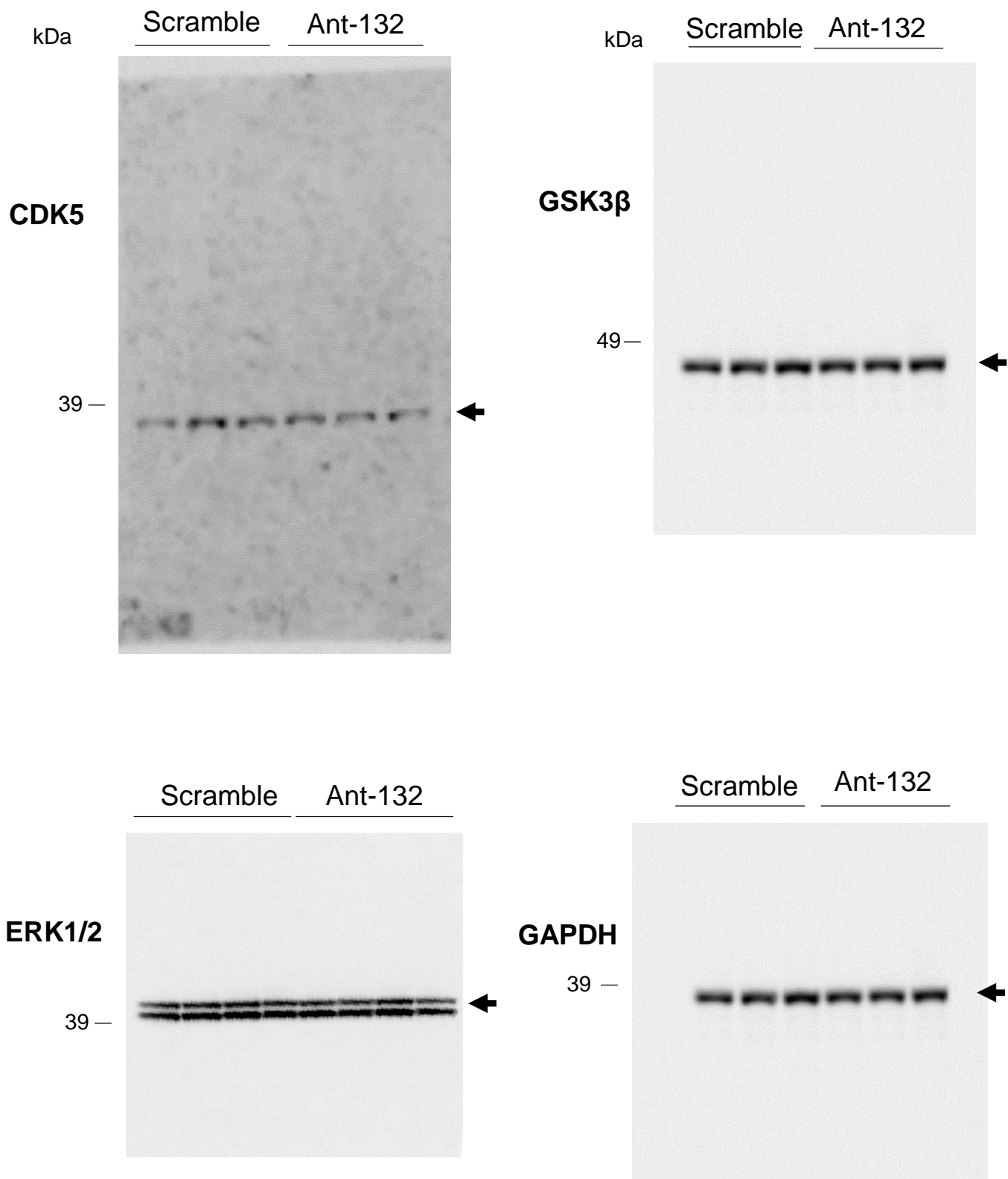

Supplement: Supplementary file 3 — Source Data for Expanded View [file EMMM-8-1005-s007.zip › Source_data_for_Expanded_View/Source_data_for_Figure_EV2.pdf]

**Figure EV3**

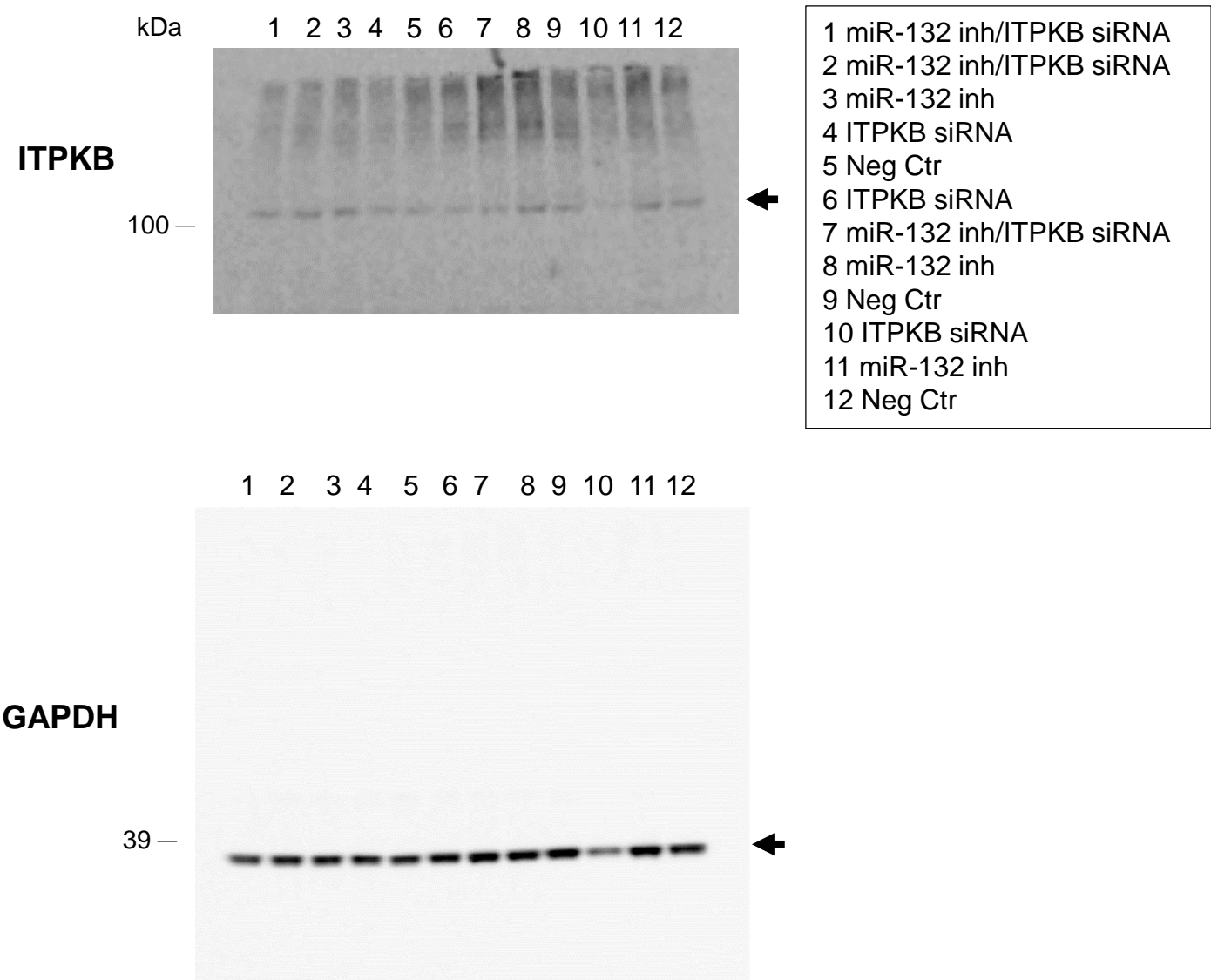

Supplement: Supplementary file 3 — Source Data for Expanded View [file EMMM-8-1005-s007.zip › Source_data_for_Expanded_View/Source_data_for_Figure_EV3.pdf]

**Figure EV4**

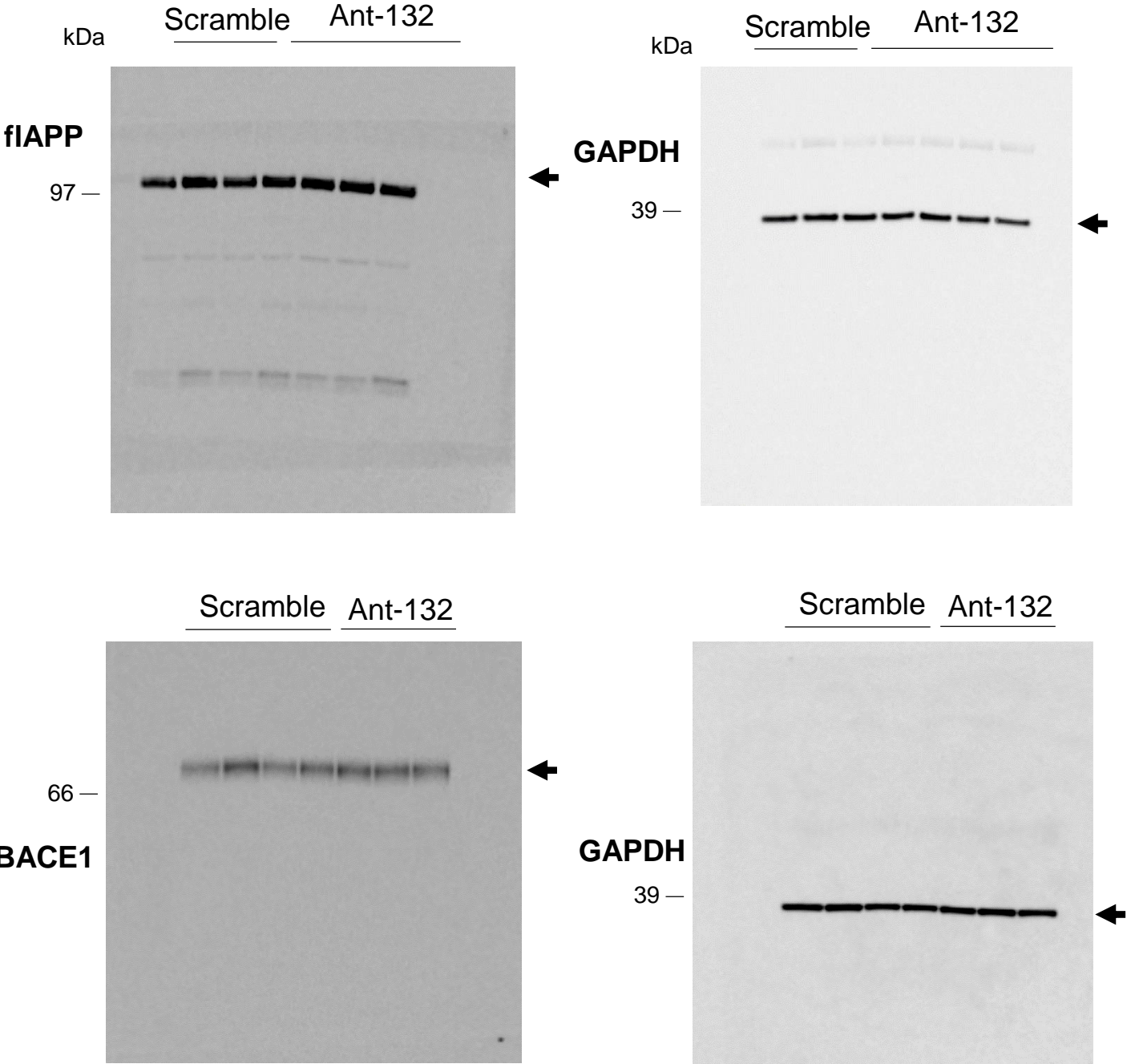

Supplement: Supplementary file 3 — Source Data for Expanded View [file EMMM-8-1005-s007.zip › Source_data_for_Expanded_View/Source_data_for_Figure_EV4.pdf]

**Figure 3**

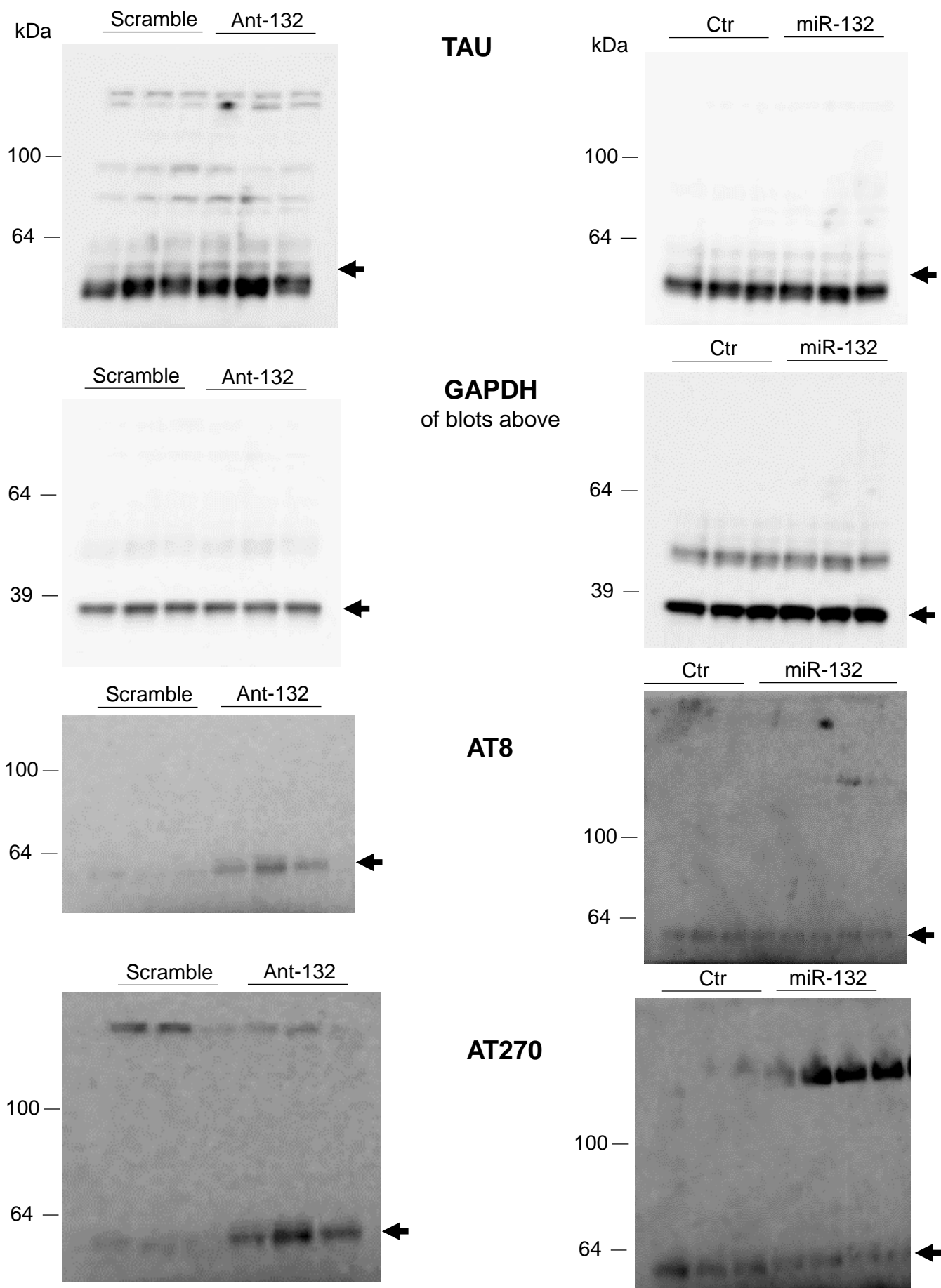

Supplement: Supplementary file 5 — Source Data for Figure 3 [file EMMM-8-1005-s003.pdf]

## Figure 5

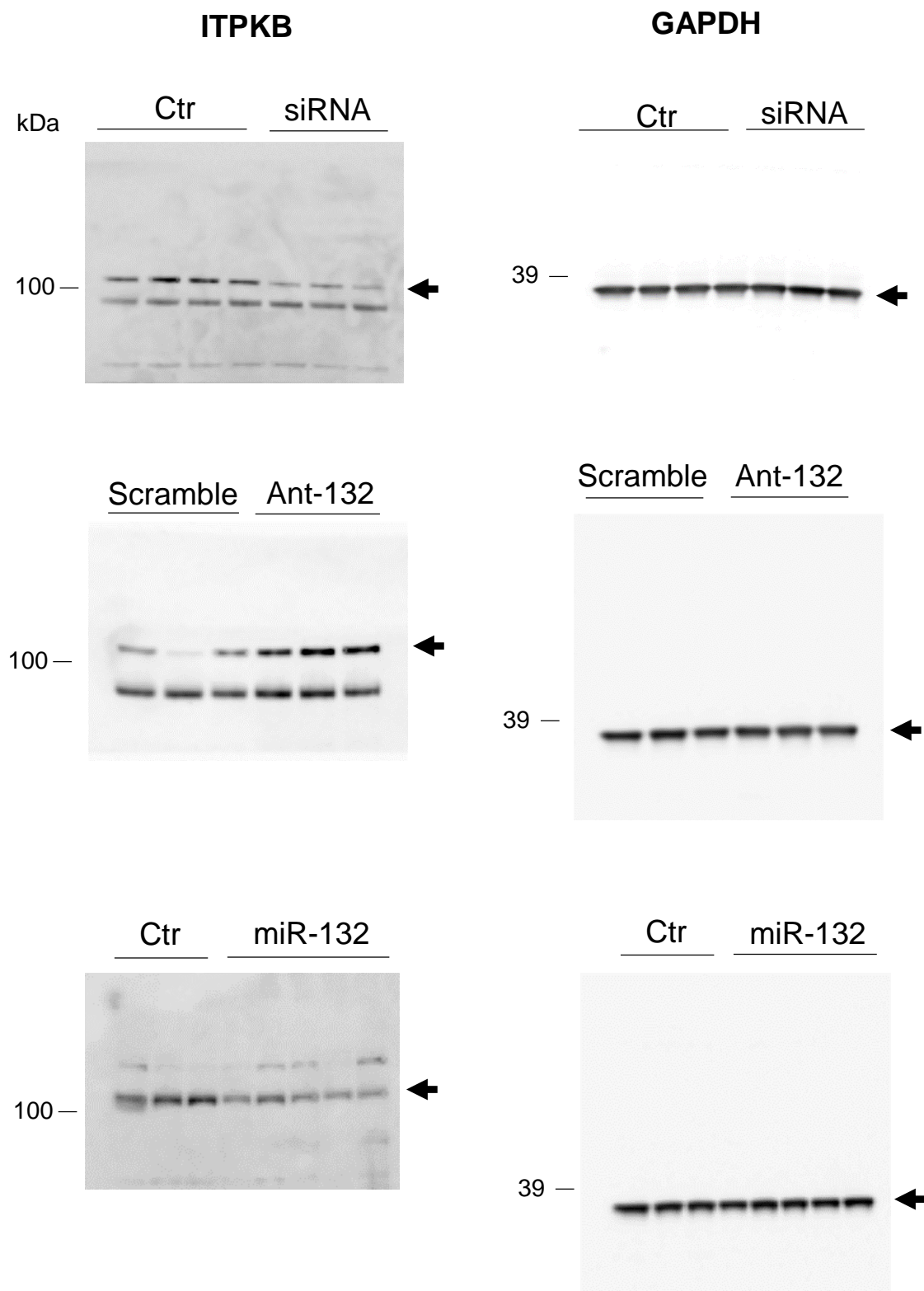

Supplement: Supplementary file 6 — Source Data for Figure 5 [file EMMM-8-1005-s004.pdf]

**Figure 6**

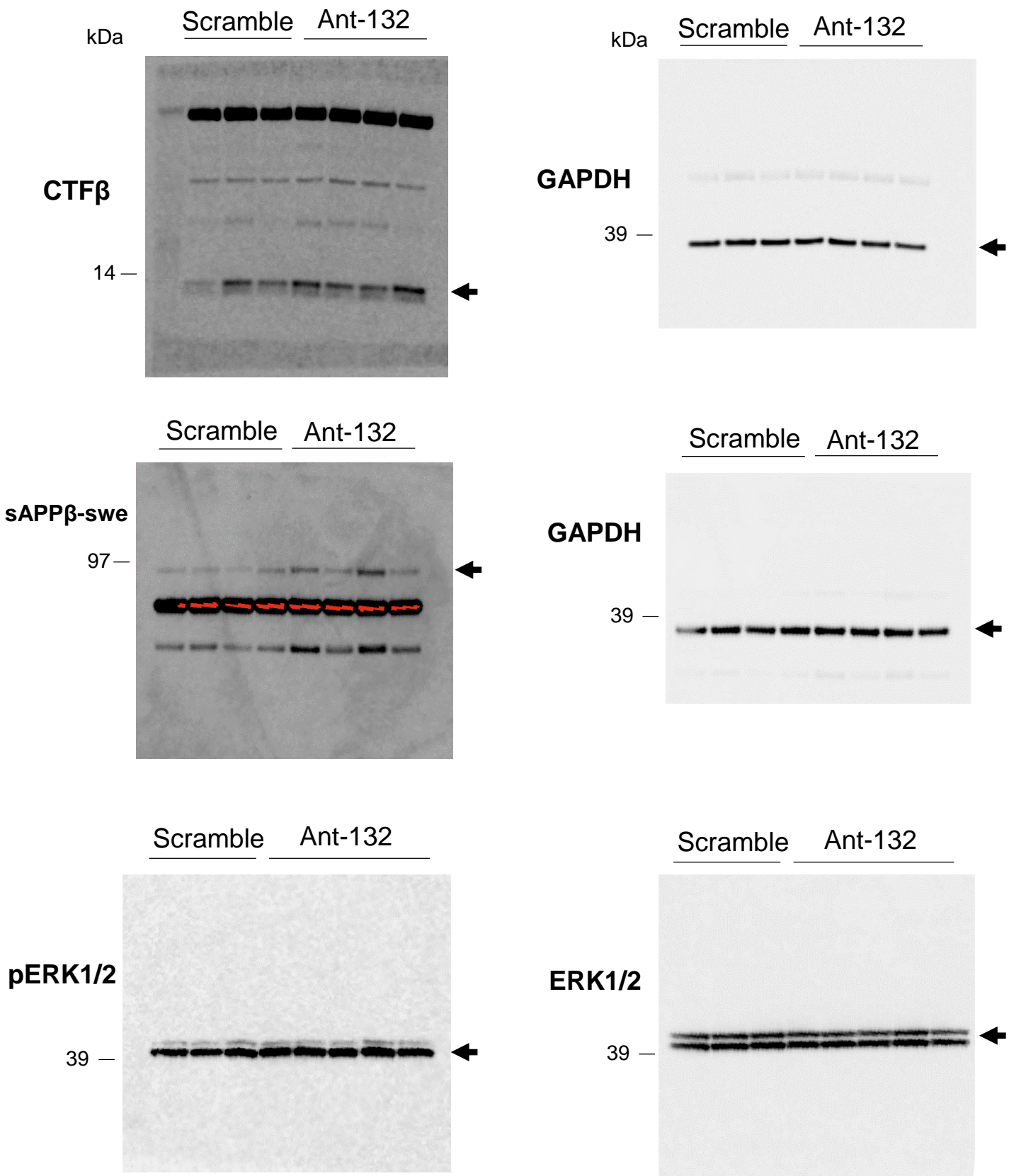

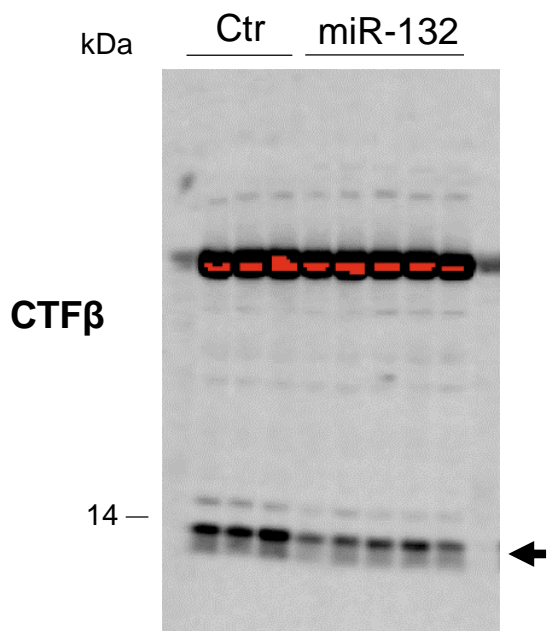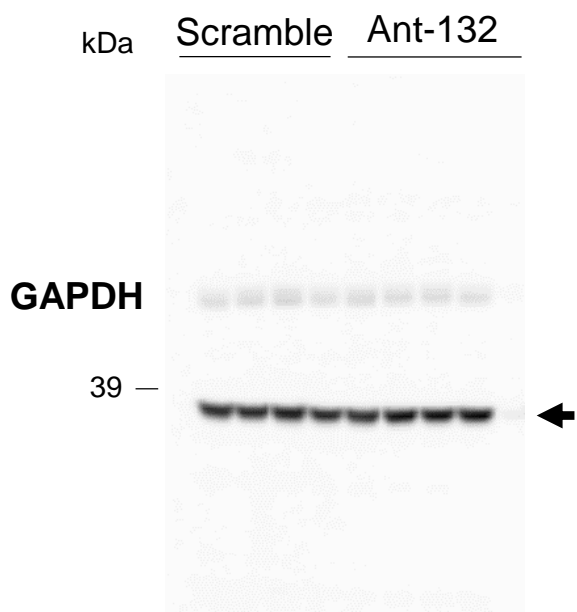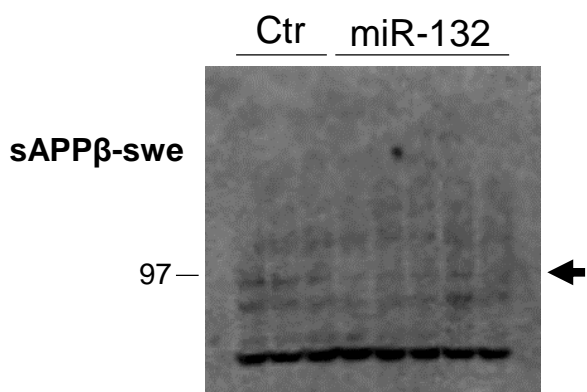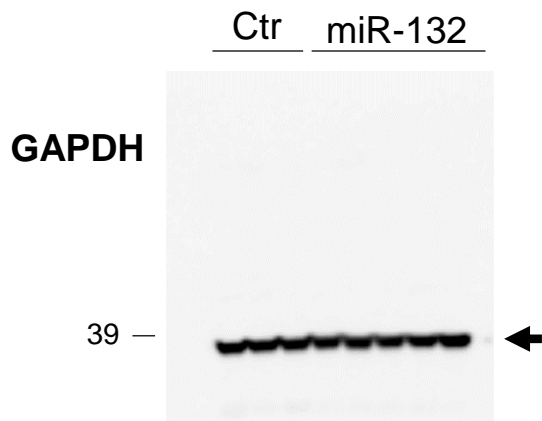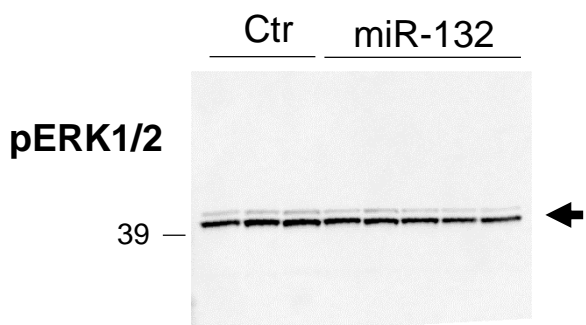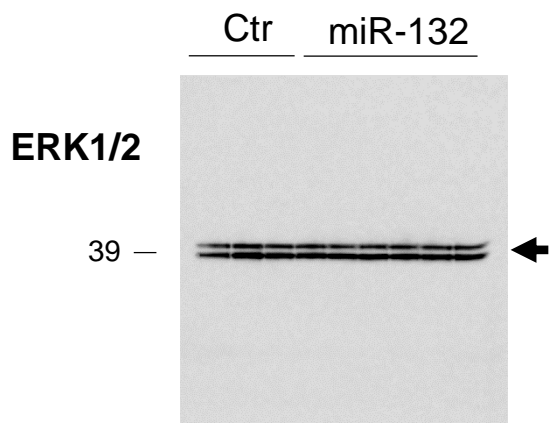

Supplement: Supplementary file 7 — Source Data for Figure 6 [file EMMM-8-1005-s005.pdf]

**Figure 8**

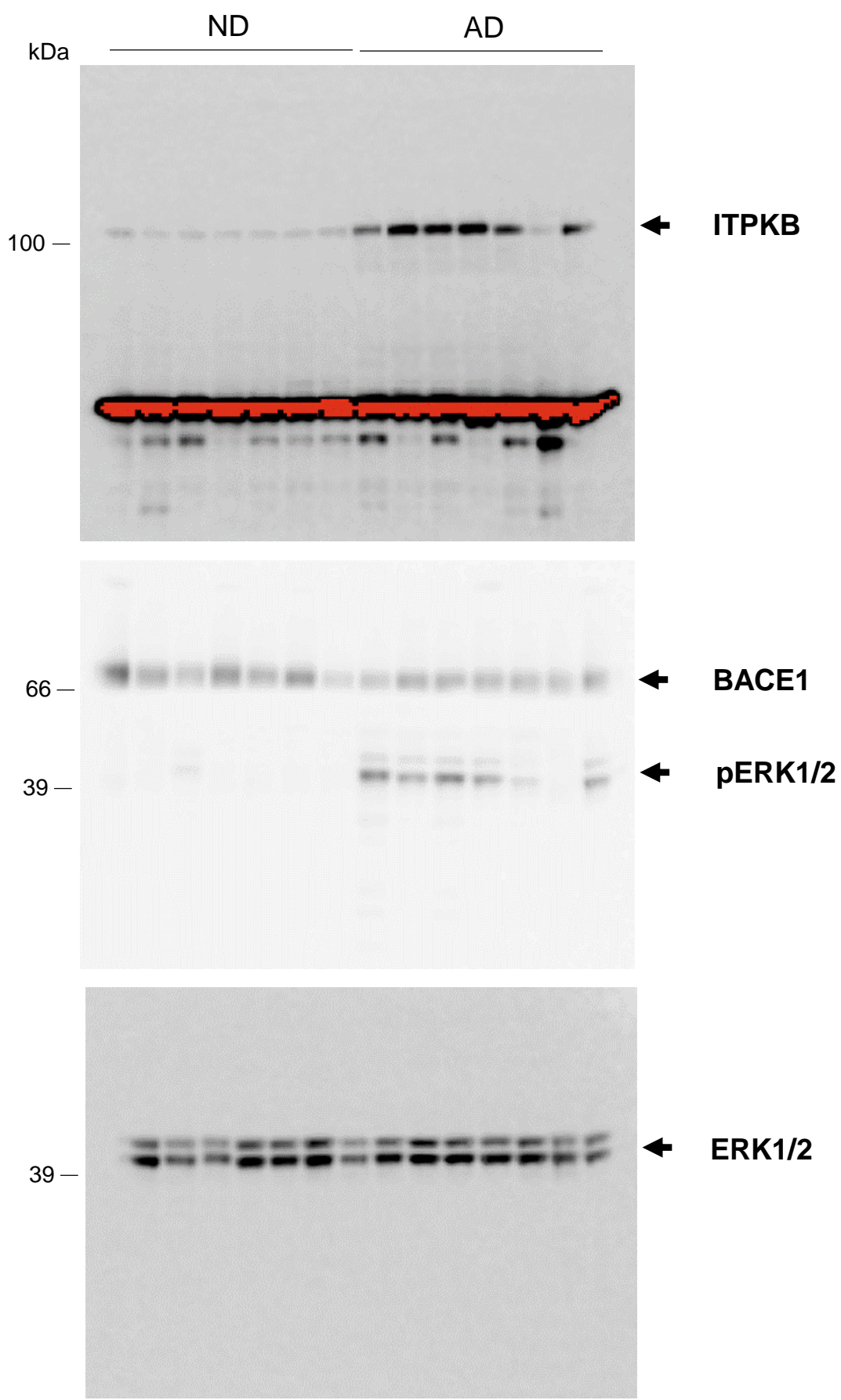

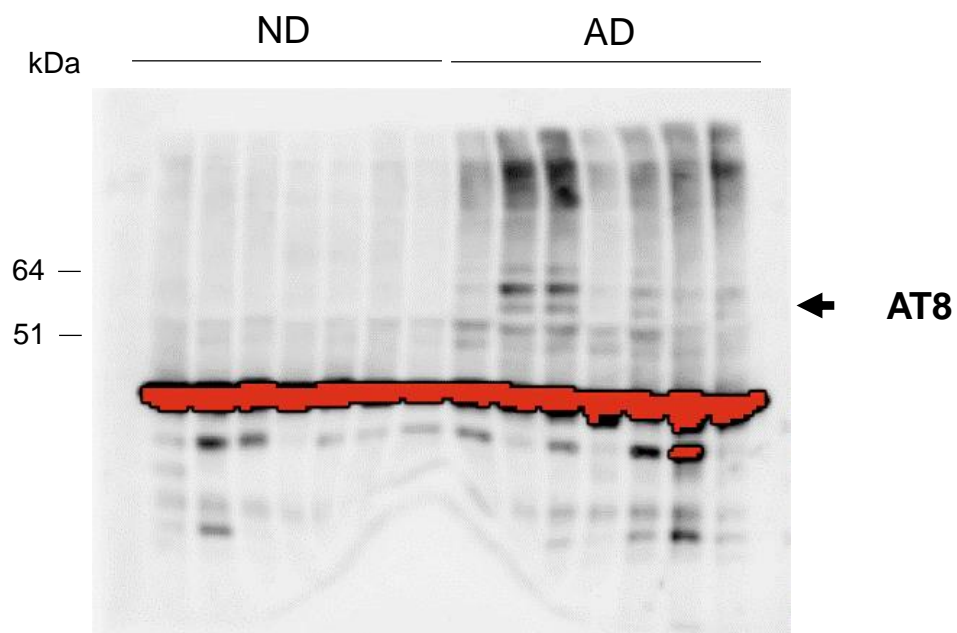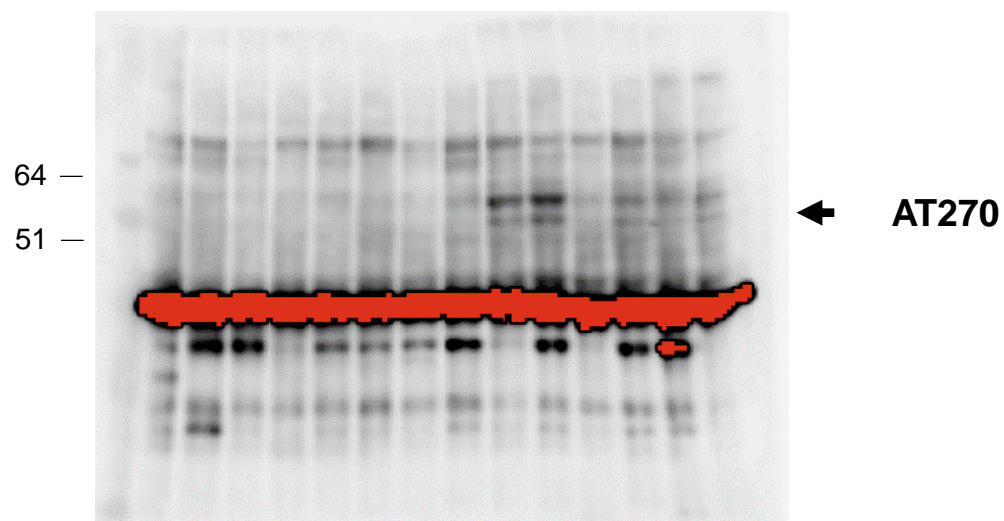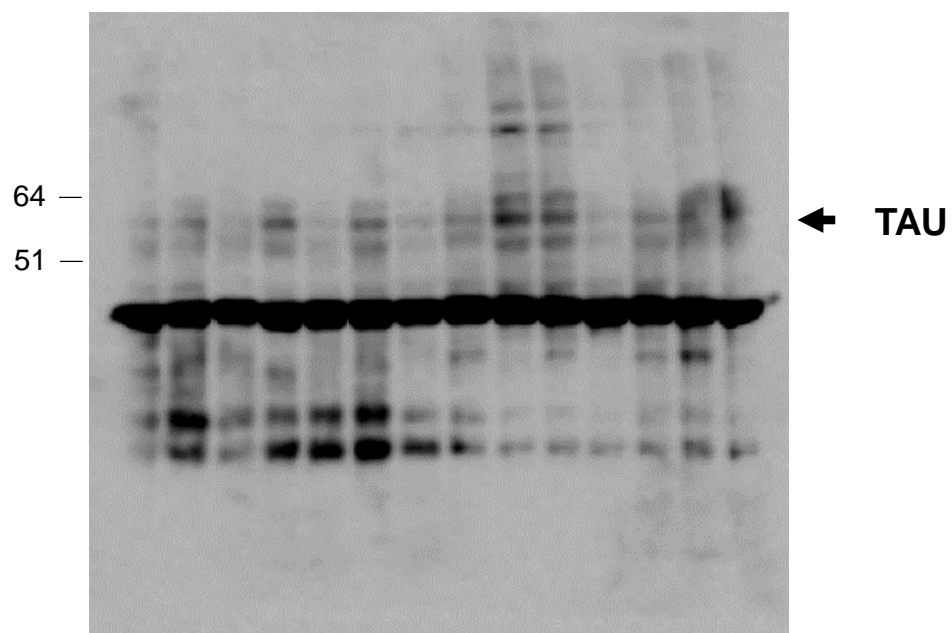

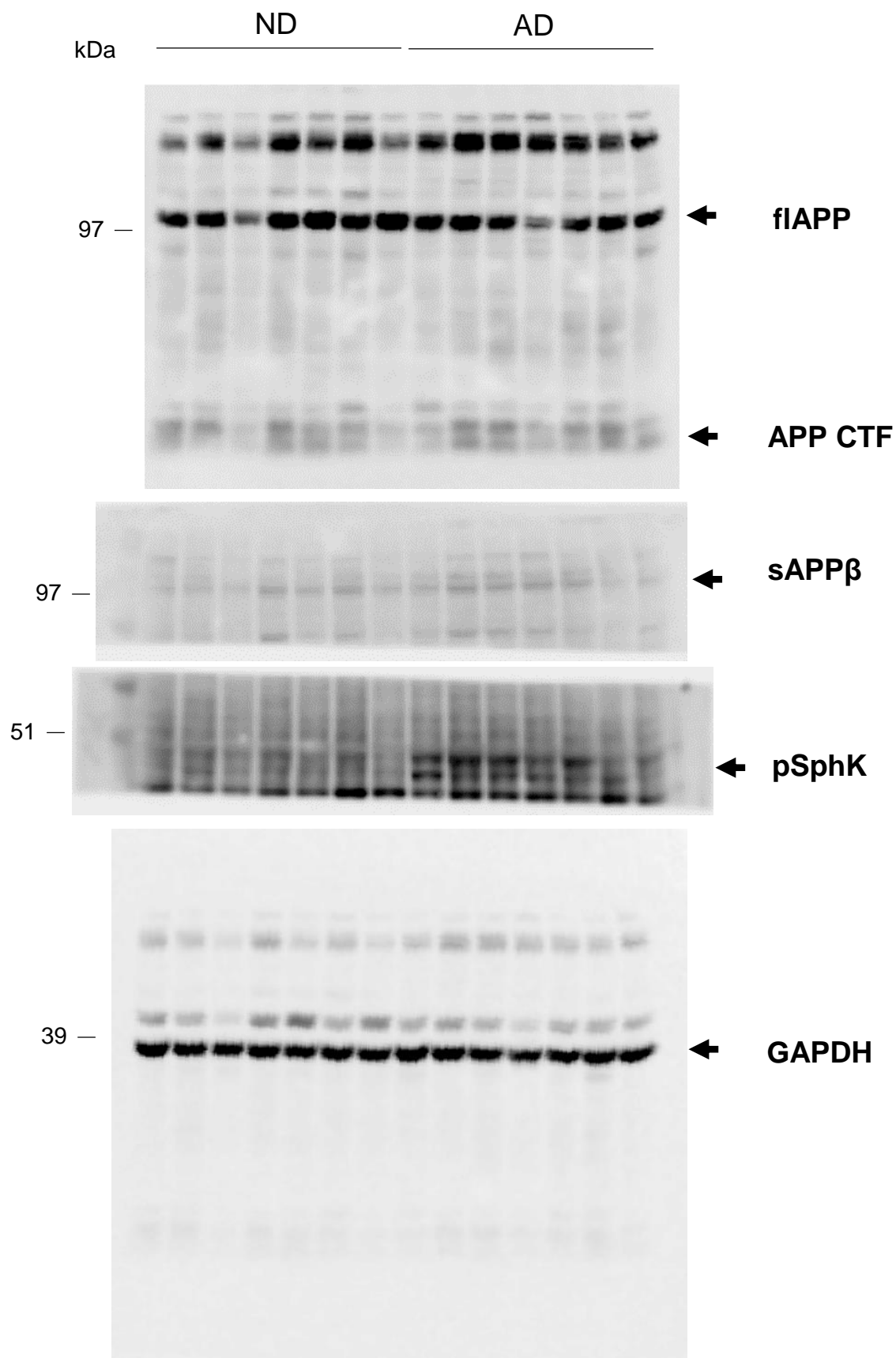

Supplement: Supplementary file 8 — Source Data for Figure 8 [file EMMM-8-1005-s006.pdf]
